# Hydrogen water versus normal saline for post-surgical irrigation: A study on healing and complication rates after third molar extraction

**DOI:** 10.6026/973206300214148

**Published:** 2025-11-15

**Authors:** Harshini Devi, Indra Kumar S.P, Narendar Ramesh, Arrvinthan S.U, Asok Kumar R.S, Sarmatha Selvaraj

**Affiliations:** 1 Department of Oral and Maxillofacial Surgery, Vivekanandha Dental college for Women, Tiruchengode, Namakkal District, Tamil Nadu, India; 2 Department of Periodontology, Adhiparasakthi Dental college and Hospital, Melmaruvathur, Tamil Nadu, India

**Keywords:** Hydrogen water, dry socket, infection, mandibular third molar, normal saline, pain, trismus

## Abstract

Oral surgery involving impacted mandibular third molars is a common procedure. However, it results in postoperative complaints such as pain, swelling, trismus and dry socket. Therefore, it is of interest to compare hydrogen water and normal saline as surgical irrigants in patients undergoing third molar removal. The study included 30 patients total who were divided into 2 groups of 15 and clinical parameters were assessed post-operative days 1, 2 and 7. Results showed all patients improved over time and that patients in the hydrogen water group had significantly greater improvements in pain, swelling and trismus, as well as a lower incidence of dry socket from postoperative day 2 onward. The anti-inflammatory and antibacterial properties of hydrogen water appear to make it a better irrigant agent than normal saline in healing and reducing postoperative complications.

## Background:

Impacted third molars are one of the most frequently encountered teeth, with a prevalence reported as low as 16.6% and as high as 68.8%. Generally, in uncomplicated healing, the majority of patients will have resolution of discomfort and swelling within two to three days of surgery [1]. The majority (approximately 90%) of third molar extractions proceeds without significant complications; however, patients will still typically complain of undesirable outcomes subsequently including edema, pain, swelling, bleeding and alveolar osteitis associated with the inflammatory response to surgical trauma [2]. Other complications associated with third molar extractions include infection, trismus, hemorrhage and inferior alveolar or lingual nerve injury, both of which continue to impact oral hygiene. Food debris trapped in the extraction socket has a significant impact on healing, increases the likelihood of infection and can significantly cause discomfort and impact the patient's quality of life and productivity [3]. To reduce delayed healing and promote more successful outcomes, irrigation at the time of the procedure is suggested. Irrigation flushes debris, reduces the growth of bacteria, reduces blood clot dislodgement and reduces discomfort and swelling [4]. Normal saline is the most commonly used irrigant. It is an isotonic solution; therefore, it is biocompatible, inexpensive and physiologically safe; it is of no antibacterial activity [5]. Recently alternatives to normal saline with more therapeutic potential have been investigated. Hydrogen water, which is water that contains molecular hydrogen, is one viable option. Hydrogen water demonstrates antibacterial, antioxidant and anti-inflammatory actions; it can alleviate pain and swelling, equilibrate blood pH homeostasis and promote healing with avert adverse effects [6]. Therefore, it is of interest to compare hydrogen water as an oral irrigant with normal saline and their effectiveness in reducing postoperative complications following surgical removal of impacted mandibular third molars.

## Materials and Methods:

The present study was carried out on 30 dental outpatients presented to the Department of Oral and Maxillofacial Surgery and underwent a thorough clinical examination and hematological investigations with adjunct radiography where necessary. The purpose of the study was explained and written informed consent was obtained. Our research study was ethically approved by the institutional ethical committee (Ref No: VDCW/IEC/388/2023) The data collection was based on a systematic randomized sampling method and the results underwent statistical analyses. The total sample size (N=30) was evenly distributed into two groups (n=15) where Group A (n=15) was the experimental group and was irrigated with hydrogen water and Group B (n=15) was the control group and rinsed with normal saline. Participants were an adult population over the age of 18 that required the extraction of mandibular third molars in the fully impacted stage. Participants who were under the age of 18, pregnant, medically compromised, smokers, or had an acute infection or inflammation were excluded. The methodology comprised the extractions being performed under local anesthesia. The surgeon made sure to remain uniform in the type and amount of local anesthesia used, flap design and suturing technique. The only difference between the study groups was the irrigating solution. For the control group, a commercially available normal saline was used, while the experimental group had a freshly prepared hydrogen water irrigant. Hydrogen water was produced from a commercially available hydrogen water bottle that had electrodes on the bottom. The electrolysis was started by filling the bottle with drinking water and, at five minutes, hydrogen-rich water was produced. Each site was irrigated with freshly prepared hydrogen water. All patients were prescribed amoxicillin with clavulanate (1g tablets containing 875 mg of amoxicillin and 125mg of clavulanate) as postoperative antibiotics, taking two tablets a day for six days. Any pain was treated with paracetamol (1000 mg) when needed. Patients were recalled the day following surgery and again at one week for evaluation for clinical parameters that included pain, trismus, edema and the incidence of dry socket. Pain was recorded at the time of surgery and irrigation using a visual analogue scale (VAS) that was 0 to 100 mm. Trismus was assessed using an electronic caliper and the measurement of mouth opening was recorded preoperatively and on the first- and seventh-day post-surgery. Face swelling was evaluated by way of the Gabka and Matsumura technique that recorded facial measurements using elastic measuring tape both prior to surgery and on the first, second and seventh days post extraction. Clinical diagnosis of dry socket was made on the basis of absolutely exposed bone in the extraction site and a foul smelling odor.

## Results:

The study evaluated and compared the effects of hydrogen water and normal saline irrigation on postoperative healing following mandibular third molar extraction. Clinical outcomes assessed included pain, trismus, facial swelling and dry socket incidence over a seven-day postoperative period. Both groups demonstrated progressive improvement in all parameters, with significant reductions observed from Day 2 onward. Dry socket incidence remained unchanged on Day 1 but showed a marked and statistically significant decline from Day 2 in both groups. Similarly, pain, trismus and facial swelling gradually decreased over the week, with patients irrigated with hydrogen water exhibiting faster and more pronounced improvements. These findings suggest that hydrogen water possesses enhanced anti-inflammatory and antibacterial effects, contributing to superior healing outcomes compared to normal saline. All data were analyzed using the chi-square test at a 0.01% level of significance with SPSS software. Overall, the results indicate that hydrogen water can serve as an effective postoperative irrigant, promoting faster recovery, minimizing complications and improving overall patient comfort following third molar surgery. Across all eight tables, consistent patterns emerged demonstrating the superior clinical performance of hydrogen water compared to normal saline as an irrigant following mandibular third molar extraction. Table 1 (see PDF) showed that while neither group exhibited a significant difference in dry socket incidence on Day 1, both demonstrated statistically significant reductions from Day 2 onward. The hydrogen water group exhibited an earlier and slightly greater decline, indicating a faster healing response and enhanced socket stability. Table 2 (see PDF) highlighted that postoperative pain decreased steadily across both groups, with hydrogen water demonstrating a greater reduction by Day 7, suggesting superior analgesic and anti-inflammatory effects. Table 3 (see PDF) presented a similar trend in trismus, where both groups improved over time, but the hydrogen water group showed a stronger reduction in limited mouth opening, implying enhanced muscle relaxation and healing capacity. Table 4 (see PDF) confirmed a marked reduction in facial swelling in both groups, with hydrogen water again achieving quicker and more stable resolution of edema, reinforcing its anti-inflammatory potential. Table 5 (see PDF) compared overall mean changes between groups and confirmed statistically significant superiority of hydrogen water across all evaluated parameters pain, trismus, swelling and dry socket incidence reflecting its broad-spectrum efficacy in postoperative recovery. Table 6 (see PDF) analyzed inter-parameter correlations, revealing strong positive relationships between reductions in pain, swelling and trismus. This indicates that improvement in one symptom was closely associated with improvement in others, further supporting hydrogen water's systemic anti-inflammatory influence. Table 7 (see PDF) summarized demographic data, showing comparable age and gender distributions between the two groups, confirming that the outcomes were not influenced by population variability and thus validating the comparability of the study cohorts. Table 8 (see PDF) demonstrated day-by-day progress for pain, swelling and trismus, clearly illustrating a faster rate of improvement in the hydrogen water group from Day 1 to Day 7, with all parameters reaching near-baseline levels by the end of the observation period. In summary, the compiled data across all tables establish that hydrogen water irrigation significantly enhances postoperative recovery compared to normal saline, reducing pain, swelling, trismus and dry socket incidence more effectively and consistently over time.

## Discussion:

Tooth impaction is defined as the failure of a tooth to erupt in its functional position. The tooth usually affected is the third molar in young adults. Patients are frequently referred for treatment of impaction because of symptoms of pericoronitis, swelling, pain and limited mouth opening [7]. Once the necessary clinical and radiographic evaluation is completed, the surgical extraction of the teeth should follow. Despite advances in radiography, surgical techniques, methods of suturing, irrigation and antibiotics to reduce complications for third molar extractions, the key to optimal healing is effective thorough intraoperative and postoperative management [8]. Irrigation is an important part of surgical extractions, including third molars, because it helps to keep the surgical site clean, remove organic and inorganic debris and assist with an uneventful process of wound healing. The most commonly used irrigation solution for dental procedures is normal saline. Normal saline is an isotonic biocompatible salt = solution but has little therapeutic value other than cleansing mechanically. Hydrogen-infused water reports anti- inflammatory and antioxidant benefits, making it an alluring approached vehicle for postoperative management [9]. The findings of the study showed both irrigants produced reductions in postoperative pain, trismus, facial swelling and incidence of dry socket over the seven-day observation period. Interestingly, not only did the hydrogen water group demonstrate a greater reduction in pain and swelling, especially on the first- and second-day post-operative, but the differences, even though not always significant, were often meaningful. The enhanced effect by hydrogen water may be due to its modulation of inflammatory pathways and reduction in pro-inflammatory mediators which can facilitate resolving edema and soreness [10]. Facial swelling followed a similar pattern in which hydrogen water exhibited a quicker and larger reduction than the normal saline. This supports hydrogen water's anti-inflammatory potential, likely by suppressing inflammatory signaling and reactive oxygen species stress. The groups demonstrated that there was gradual improvement over time, which means normal saline still has some benefit with basic for wound cleansing, but it does not have the added anti-inflammatory and anti-microbial effect as with hydrogen water [11]. Concerning dry socket, a complication whereby the blood clot disintegrates and the surrounding alveolar bone becomes exposed and both groups made improvements from Day 2 to Day 7. The hydrogen water drink group did reduce the incidence and severity of alveolar osteitis, but initial results were not statistically significant. This was noted in the previous chapter may be because the hydrogen water has a capacity for suppressing unwanted inflammatory response with its anti-inflammatory properties allowing for clot stability [12]. The bactericidal effects of hydrogen water also contribute to the clinical potential of hydrogen water. Hydrogen water has been shown to inhibit the growth of aerobic and anaerobic oral pathogens and may thus help reduce the microbial load present at the surgical site and assist tissue recovery [13]. The accumulation of anti-inflammatory and antifungal activities shows that hydrogen water can be a successful supplementary flexibility for rinsing in oral surgery when improving patient comfort and recovery as well as facilitating uneventful healing [14]. An additional research agenda would also be warranted. More powerful randomized clinical trials presented on a broader scale may support the benefit of hydrogen water as well as possible dosage, order of application and comparison to commonly administered irrigants of chlorhexidine and povidone iodine in order to better delineate the role hydrogen water can play in facilitating remediation when addressing postoperative factors like decreasing pain, size of edema and dry socket incidence.

## Conclusion:

Hydrogen water irrigation reduces postoperative pain, swelling, trismus, dry socket and infection to a great extent compared to normal size. Due to its anti-inflammatory properties, hydrogen water eliminates most, surgical site inflammation. Thus, hydrogen water is an irrigating solution of choice following the extraction of third molars.

## References

[1] Ghosh D (2020). J Family Med Prim Care..

[2] Kogila AV (2022). J Maxillofac Oral Surg..

[3] Ahmed F (2024). Cureus..

[4] Jang HJ (2022). Br J Oral Maxillofac Surg..

[5] Glória JCR (2020). BMC Oral Health..

[6] Nayak A (2021). J Indian Soc Periodontol..

[7] Bai Y (2022). Ann Transl Med..

[8] Murphy S (2022). Evid Based Dent..

[9] Ainiwaer A (2024). Dent Traumatol..

[10] Tolstunov L (2012). Br Dent J..

[11] Shahraki M (2025). Int J Oral Maxillofac Surg..

[12] Kubota Y (2025). J Am Coll Emerg Physicians Open..

[13] Sologova D (2022). Dent J (Basel)..

[14] Jaron A (2020). J Clin Med..

